# Supplementary material for: Geometric and topological characterization of the cytoarchitecture of islets of Langerhans
Source: PLoS Comput Biol. 2023 Nov 9;19(11):e1011617. doi: 10.1371/journal.pcbi.1011617 (PMC10662755; doi:10.1371/journal.pcbi.1011617)
Supplement: S1 Appendix — An illustration of dimension-1 PH computed for a point-cloud and intuitive interpretation of the results. (PDF) [file pcbi.1011617.s001.pdf]

1 Illustrating persistent homology applied to a point-cloud

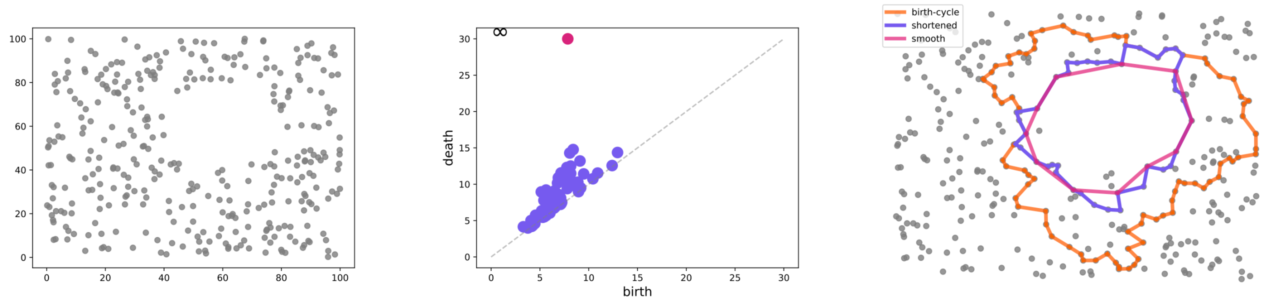

(A) (B) (C)

Figure 1: Illustrating persistent homology to compute robust topological features in a discrete data set. (A) Discrete set of points. (B) Persistence diagram shows one feature stands out with high persistence (death - birth). (C) Our algorithms find tight representative bondaries after shortening and smoothing algorithms.

Fig. 1A shows an example of a discrete set of points or a point-cloud. It has many gaps and holes, but one stands out as a larger hole compared to others. Dimension-1 persistent homology (PH) computes birth and death of holes across different spatial scales. This information is plotted as a persistence diagram (PD). Fig. 1B shows dimension-1 PD computed for the example in Fig. 1A. Persistence of a hole is defined as the difference between its death and birth. Those with higher persistence are more robust to noise in the data set. There is one feature (marked in red) with relatively higher dimension-1 persistence, as expected. Fig. 1C shows multiple possible representative boundaries computed around the feature with maximum persistence. The shorter ones, that wrap more precisely around the feature, were computed using algorithms developed in [1].

References

1. Aggarwal M, Periwal V. Tight basis cycle representatives for persistent homology of large biological data sets. PLOS Computational Biology. 2023;19(5):1–23. doi:10.1371/journal.pcbi.1010341.
