# Supplementary material for: Geometric and topological characterization of the cytoarchitecture of islets of Langerhans
Source: PLoS Comput Biol. 2023 Nov 9;19(11):e1011617. doi: 10.1371/journal.pcbi.1011617 (PMC10662755; doi:10.1371/journal.pcbi.1011617)
Supplement: S1 Table — (PDF) [file pcbi.1011617.s002.pdf]

S1 Table

| KS-significance test p-values for all islets | Stage 0 | Stage 1 | Stage 2 |
| --- | --- | --- | --- |
| **Stage 1** | 5E-22 | | |
| **Stage 2** | 3E-186 | 1E-89 | |
| **Stage 3** | 0 | 5E-221 | 7E-20 |
| | | | |
| | **Diabetic** | | |
| **Control** | 2E-08 | | |
