# Supplementary material for: Geometric and topological characterization of the cytoarchitecture of islets of Langerhans
Source: PLoS Comput Biol. 2023 Nov 9;19(11):e1011617. doi: 10.1371/journal.pcbi.1011617 (PMC10662755; doi:10.1371/journal.pcbi.1011617)
Supplement: S2 Table — Upper diagonal matrix shows comparison for islets with a NS β-component in an αδ-cycle. Lower diagonal matrix shows comparison for islets with a NS αδ-component in a β-cycle. (PDF) [file pcbi.1011617.s003.pdf]

S2 Table

KS-significance test p-values for islets with mantle around NS component	Stage 0	Stage 1	Stage 2	Stage 3
**Stage 0**		0.0001	0.0006	0.0001
**Stage 1**	0.007		0.0012	1E-10
**Stage 2**	9E-15	7E-10		1E-05
**Stage 3**	5E-18	9E-15	0.02	
	**Control**	**Diabetic**		
**Control**		0.0003		
**Diabetic**	0.011			
