# Supplementary material for: Geometric and topological characterization of the cytoarchitecture of islets of Langerhans
Source: PLoS Comput Biol. 2023 Nov 9;19(11):e1011617. doi: 10.1371/journal.pcbi.1011617 (PMC10662755; doi:10.1371/journal.pcbi.1011617)
Supplement: S3 Table — (PDF) [file pcbi.1011617.s004.pdf]

S3 Table

Geom. vs. PH KS-significance test p-values	alpha-delta mantle around NS beta component	beta-mantle around NS alpha-delta component
**Stage 0**	1	1
**Stage 1**	1	1
**Stage 2**	1	1
**Stage 3**	1	0.99
**Control**	1	1
**Diabetic**	1	1
