# Supplementary material for: Geometric and topological characterization of the cytoarchitecture of islets of Langerhans
Source: PLoS Comput Biol. 2023 Nov 9;19(11):e1011617. doi: 10.1371/journal.pcbi.1011617 (PMC10662755; doi:10.1371/journal.pcbi.1011617)
Supplement: S4 Table — (PDF) [file pcbi.1011617.s005.pdf]

Table 1

ID	Age	Sex
D1	38	F
D2	42	M
D3	65	F
D4	66	F
D5	67	F
D6	67	F
D7	71	F
D8	71	M
D9	72	F
D10	73	M
D11	75	F
D12	81	M
C1	15	M
C2	21	M
C3	24	M
C4	41	F
C5	45	F
C6	47	M
C7	51	F
C8	51	M
C9	53	M
C10	63	F
C11	63	F
C12	68	M
C13	73	M
C14	81	M
