# Supplementary figures and images for: Geometric and topological characterization of the cytoarchitecture of islets of Langerhans
Source: PLoS Comput Biol. 2023 Nov 9;19(11):e1011617. doi: 10.1371/journal.pcbi.1011617 (PMC10662755; doi:10.1371/journal.pcbi.1011617)

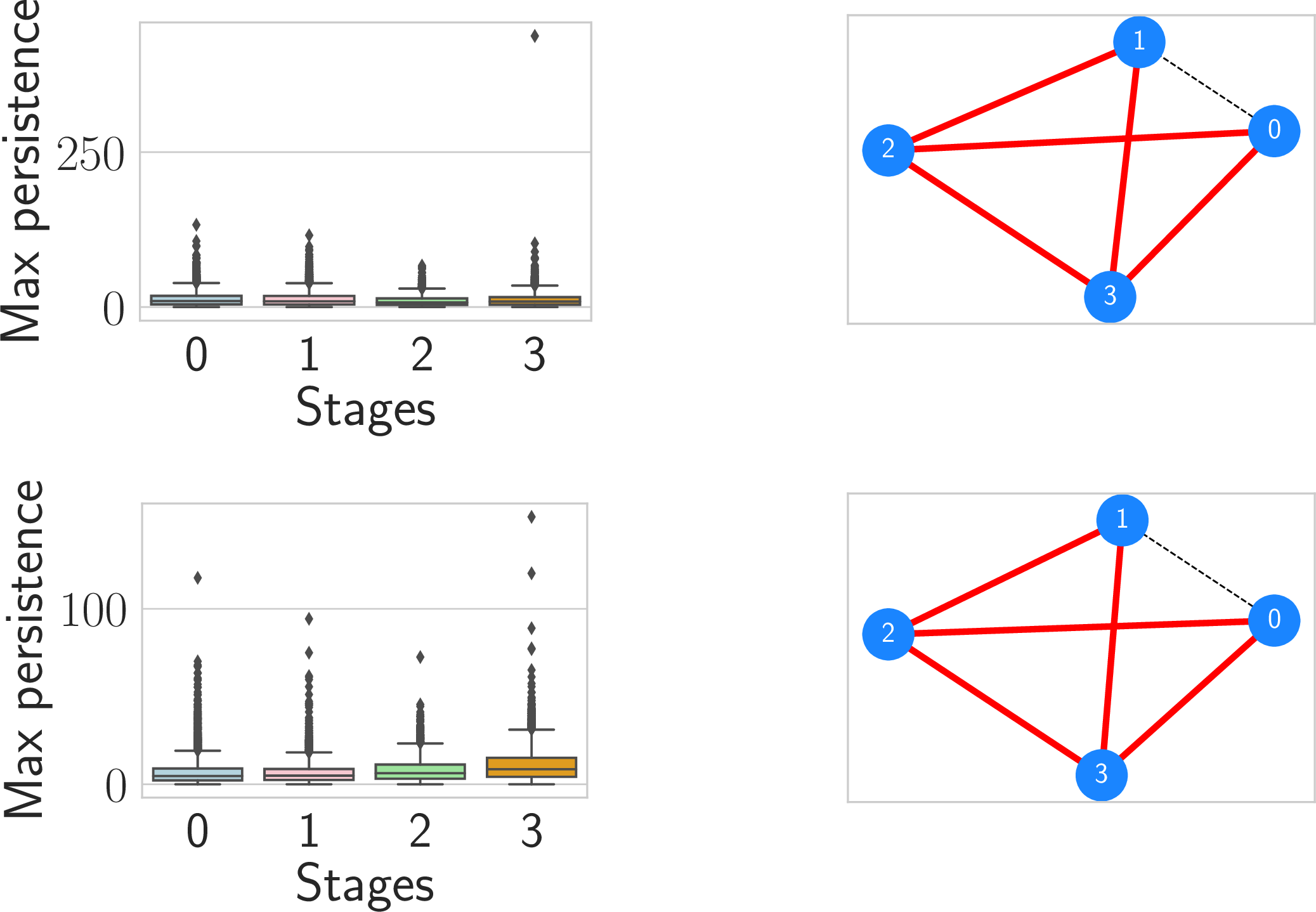

Supplement: S1 Fig — Top row is αδ-cells. Bottom row is β-cells. Left column shows box plots. Right column shows the significance results from pairwise Mann-Whitney U tests. Black dotted edges represent p-value >0.05 and solid thick red edges represent p-value <0.001. Only stages 0 and 1 are not significantly different. (TIF) [file pcbi.1011617.s006.tif]

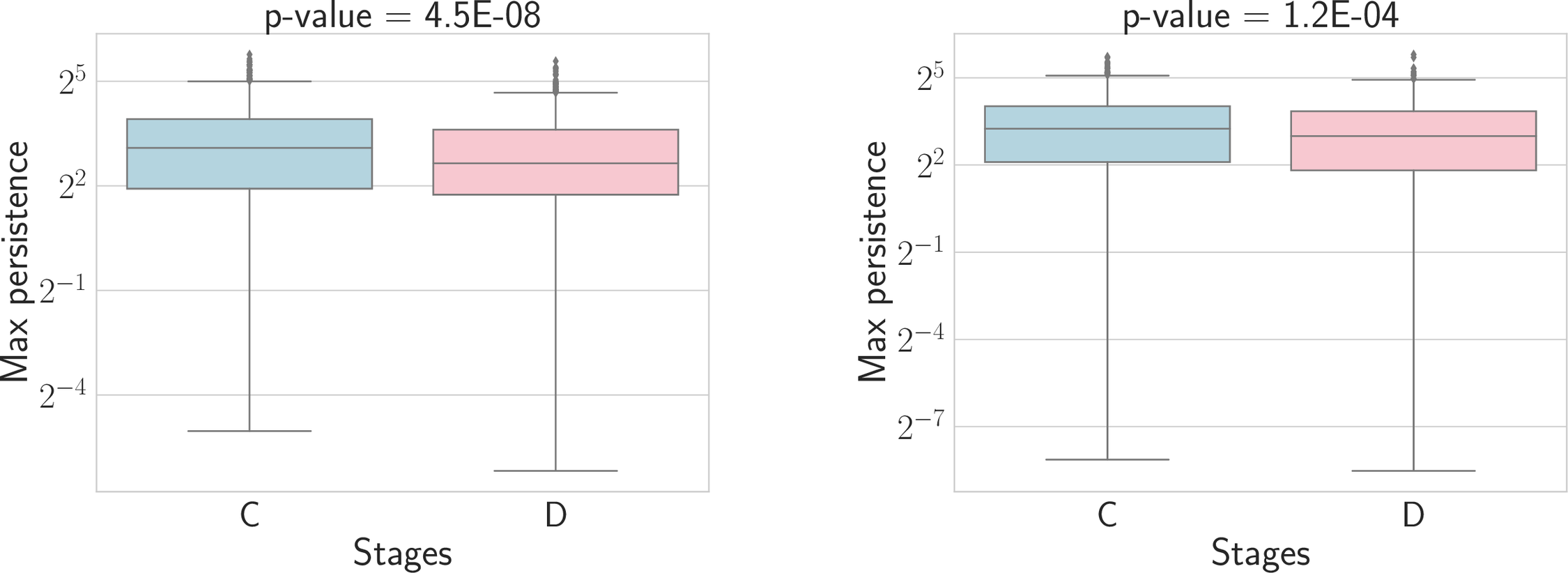

Supplement: S2 Fig — Left column is for topology of αδ-cells and right column is for topology of β-cells. Both are significantly different since p-value <0.05. (TIF) [file pcbi.1011617.s007.tif]

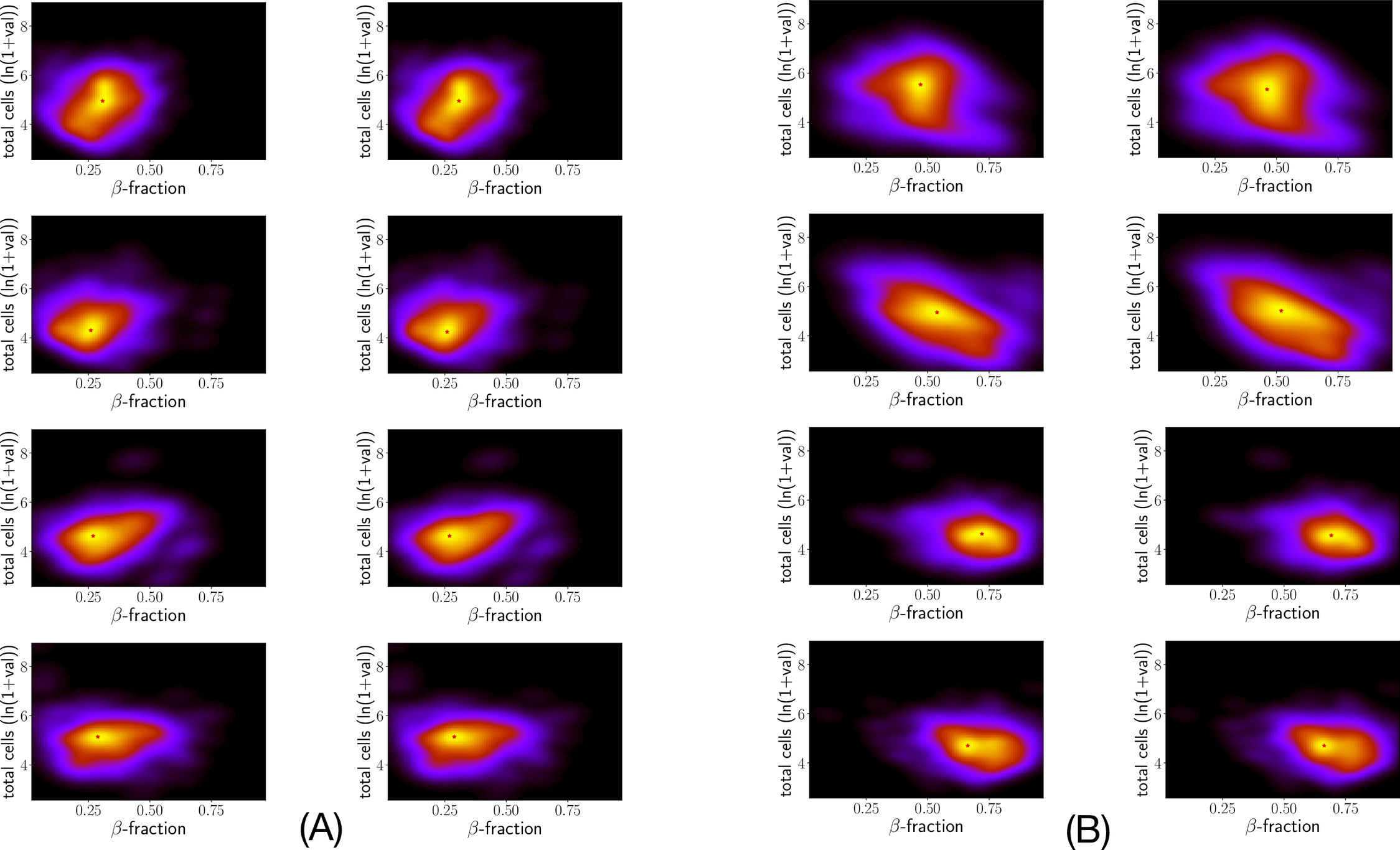

Supplement: S3 Fig — KDEs of islets with (A) αδ-cycles around at least one NS β-component, and (B) β-cycles around at least one NS αδ-component. In each panel, left column is for geometric cycles and right column is PH-cycles. Rows are stages are 0 to 3 from top to bottom. (TIF) [file pcbi.1011617.s008.tif]

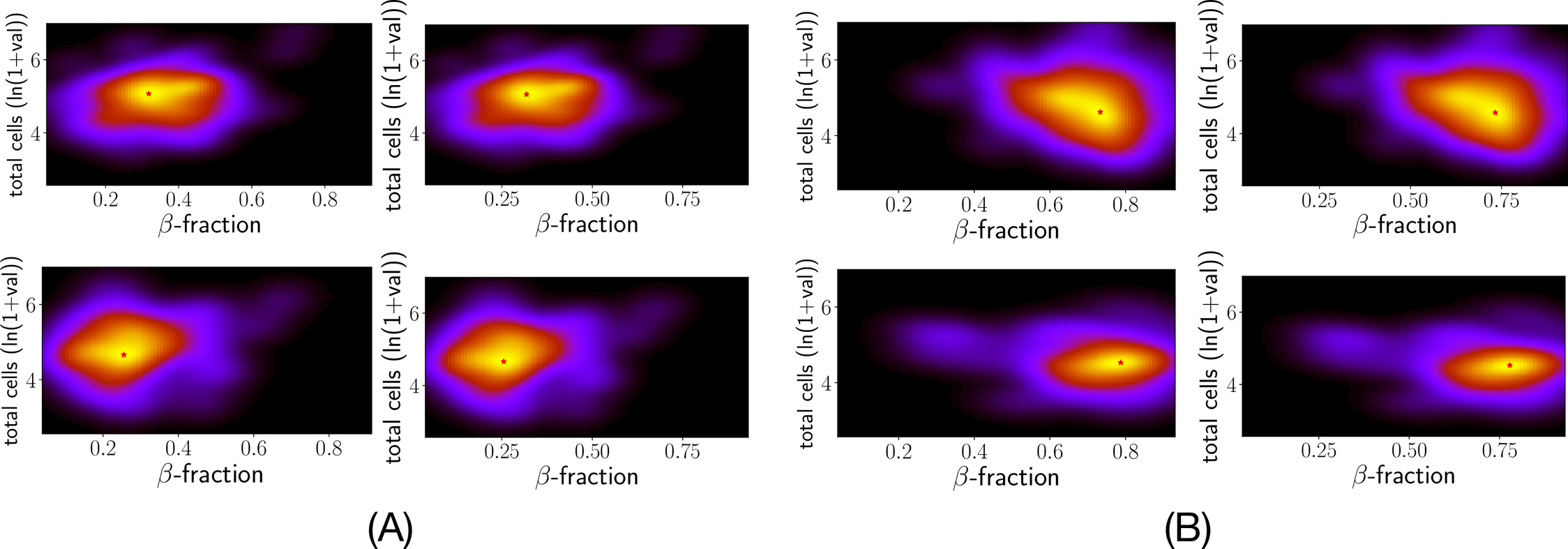

Supplement: S4 Fig — KDEs of islets with (A) αδ-cycles around at least one NS β-component, and (B) β-cycles around at least one NS αδ-component. In each panel, left column is for geometric cycles and right column is PH-cycles. Top row is non-diabetic or control and bottom row is for diabetic subjects. (TIF) [file pcbi.1011617.s009.tif]

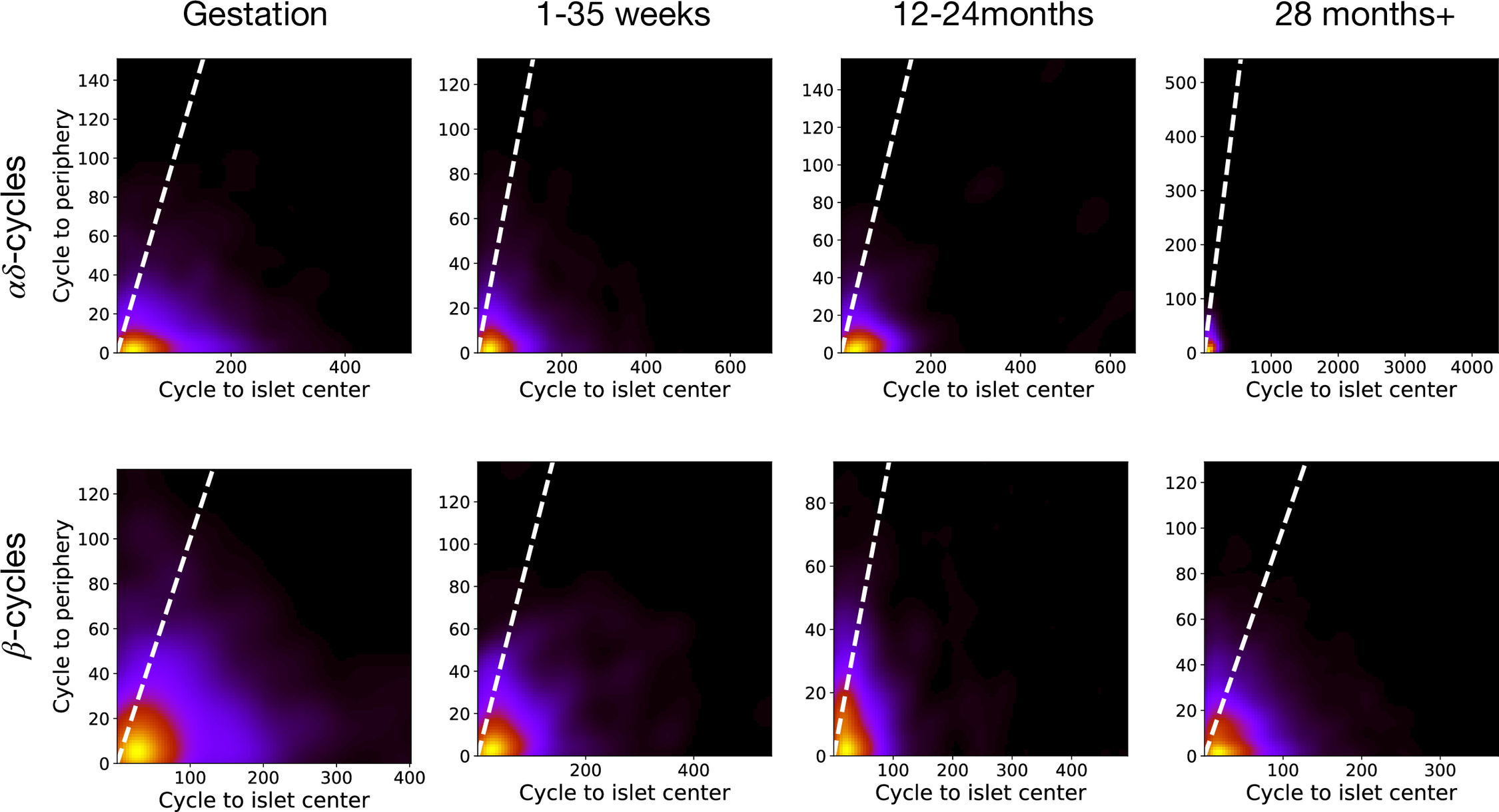

Supplement: S5 Fig — (TIF) [file pcbi.1011617.s010.tif]

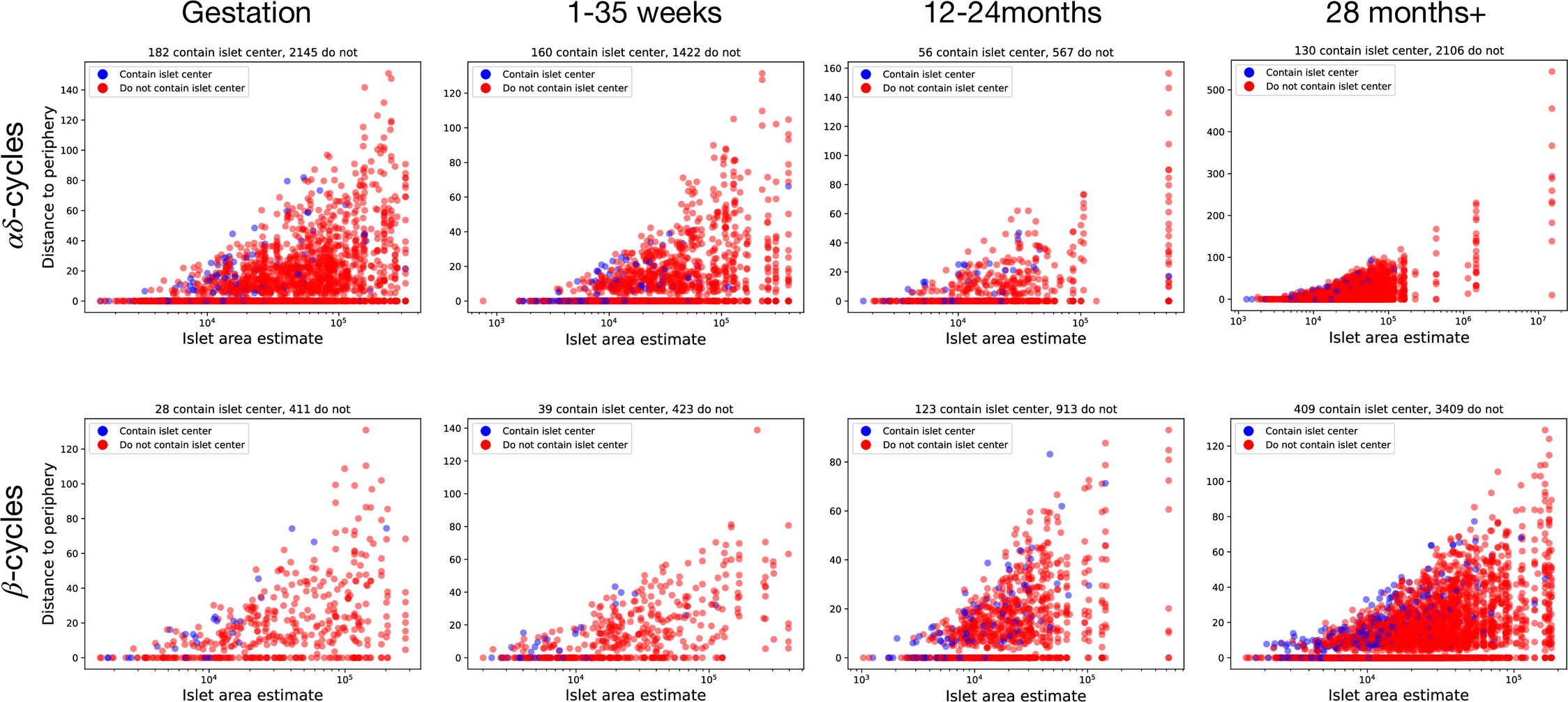

Supplement: S6 Fig — (TIF) [file pcbi.1011617.s011.tif]

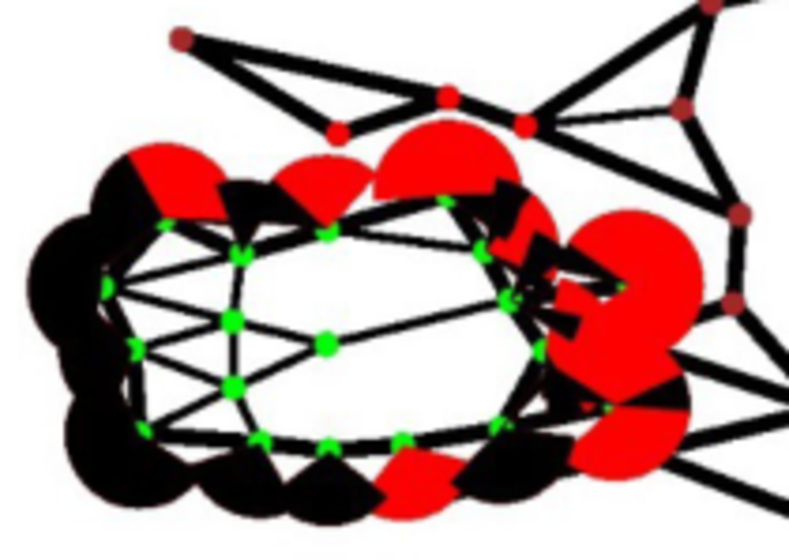

Supplement: S7 Fig — A component of β-cells (green) is partially surrounded by αδ-cells (small red points). The red arcs around the β-cells show the region surrounded and the black arcs show the region not surrounded. (TIF) [file pcbi.1011617.s012.tif]

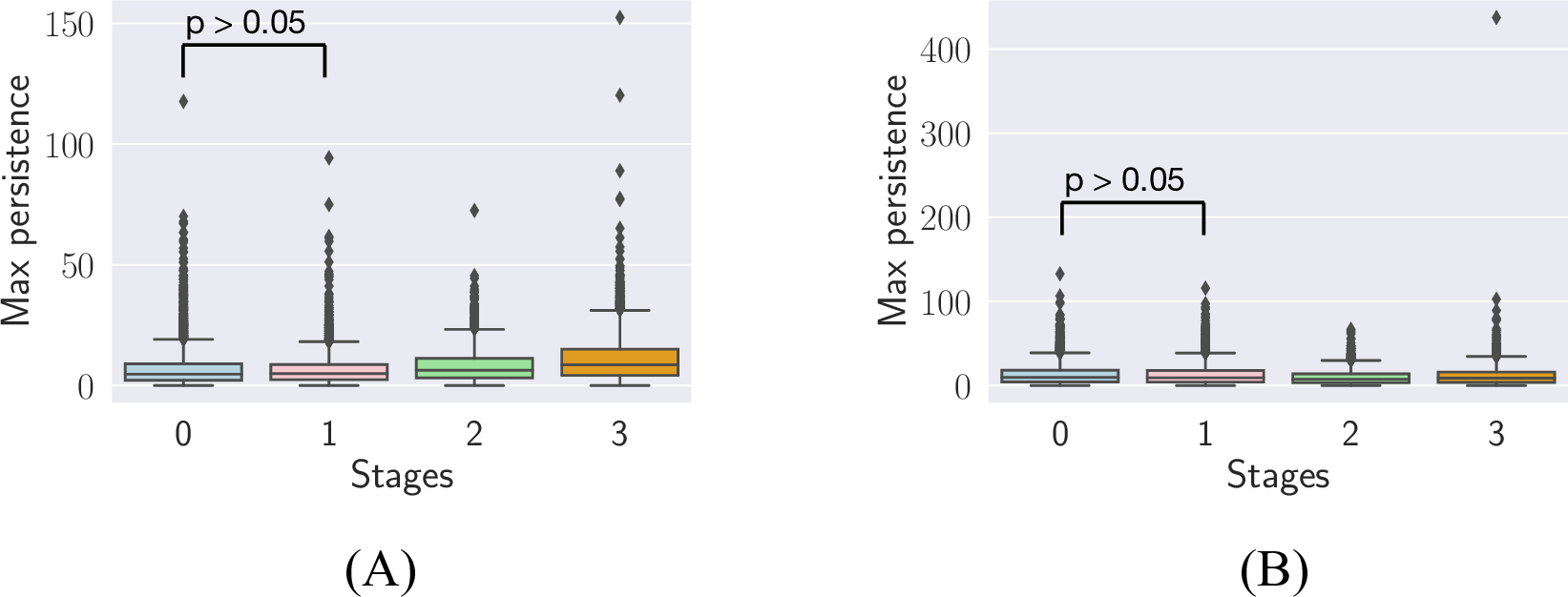

Supplement: S8 Fig — Except for developmental stages 0 and 1, all other pairwise comparisons showed significant difference (p ≪ 0.05) using Mann-Whitney U test. (TIF) [file pcbi.1011617.s013.tif]

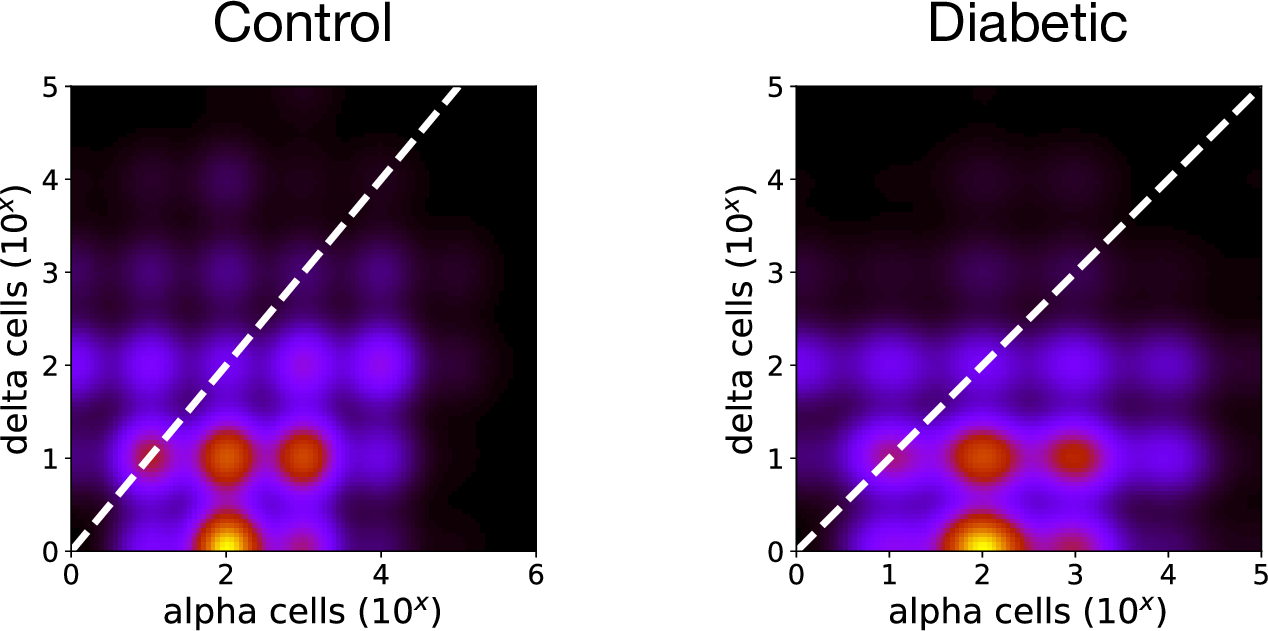

Supplement: S9 Fig — (TIF) [file pcbi.1011617.s014.tif]

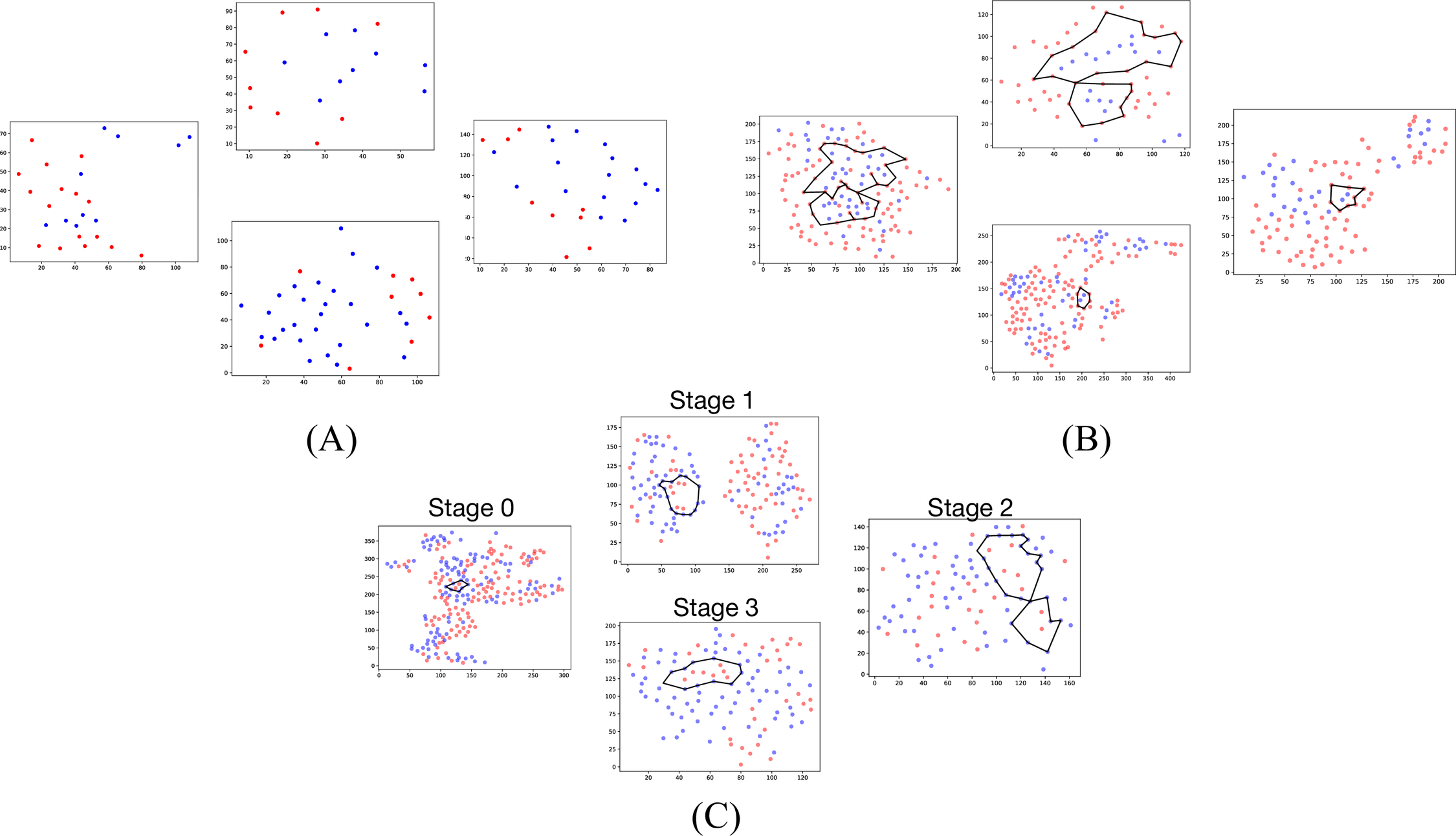

Supplement: S10 Fig — Examples of sections with characteristics close to the peak in KDE for (A) all islets, (B) islets with at least one NS β-component surrounded by a cycle, and (C) islets with at least one NS αδ-component surrounded by a cycle. (TIF) [file pcbi.1011617.s015.tif]

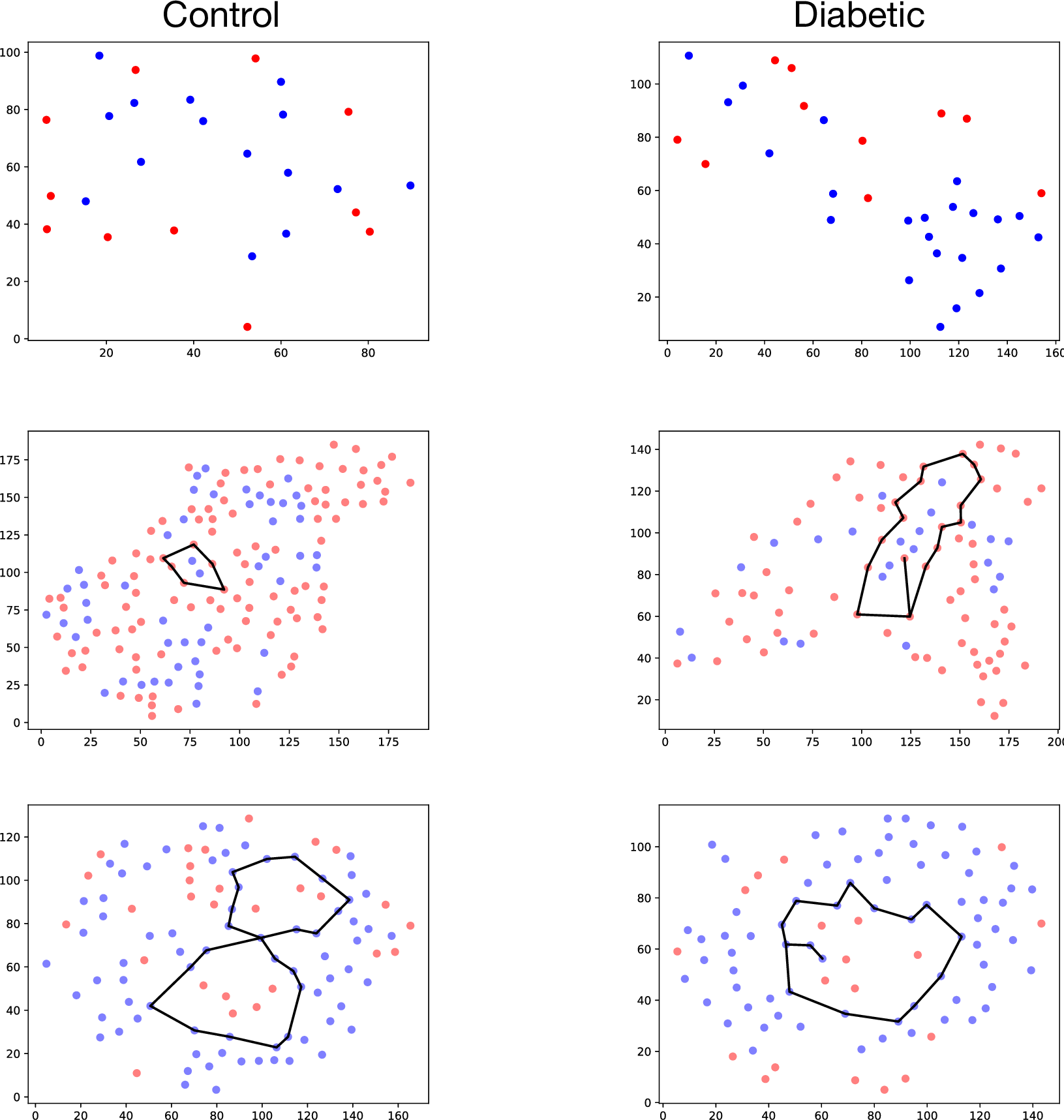

Supplement: S11 Fig — (TIF) [file pcbi.1011617.s016.tif]
