# Supplementary material for: Geometric and topological characterization of the cytoarchitecture of islets of Langerhans
Source: PLoS Comput Biol. 2023 Nov 9;19(11):e1011617. doi: 10.1371/journal.pcbi.1011617 (PMC10662755; doi:10.1371/journal.pcbi.1011617)
Supplement: Attachment — Submitted filename: PH Islets Review PLOS Comp Bio.docx [file pcbi.1011617.s017.docx]

Geometric and Topological Characterization of the Cytoarchitecture of Islets of Langerhans

The article deals with a geometric technique and persistent homology (PH) for the investigation of the cytoarchitecture of the islets of Langerhans.

Minor concerns:

1. PH is described as computing topological invariants “from networks,” which is incorrect. PH computes topological invariants from a simplicial complex upon a point cloud of data.
2. There are repeated references to PH uncovering “non-local robust network features,” but this is never defined. It is unclear what “non-local” refers to and what sort of robustness is used.
3. Their explanation of PH in Methods discusses vertices, edges, and triangles, but makes no mention of higher-dimensional structures that may appear in the simplicial complex. These structures can appear even in lower-dimensional data due to the construction of the simplicial complex. Additionally, the words used (borrowed from network theory and geometry) are not the proper vocabulary for these topological objects. In the context of PH, these are the 0-simplex, 1-simplex, 2-simplex, etc.
4. The description of the PH methods are very technical and difficult to parse. I would suggest referring to existing explanations of PH in the literature to see if it can be simplified for a computational biology audience.

Major concerns:

1. PH is computed using software developed by one of the authors called Dory. While information regarding Dory has been uploaded to arXiv, the software has not been published in a peer-reviewed setting. This raises questions about the accuracy of Dory compared to published software like Ripser, giotto-tda, javaPlex, etc.
2. There is a claim that the authors have developed “technical tools for finding tight representatives for topological features that can be explicitly localized for visualization.” It is not clear to me what this means. The discussion further claims that the authors’ method “explicitly give[s] the locations of the actual topological features in the image,” but no such method is described in the Methods section or described in the Results.
3. “Mantles” are computed using geometric and topological techniques but are not defined.
4. Figure 5B features two separate line graphs consisting of two datapoints each (control vs. diabetic), which is not an appropriate visualization for this data. There is no statistical test to compare the percentiles between control and diabetic.
5. A pair distribution function is used for the distance computation in PH. It’s not immediately obvious to me that the function is a metric. The construction of a simplicial complex requires a metric. Please provide a reference or proof to establish this.
6. The geometric method introduced is complex and the significance of each step is not explained. It is claimed that this method detects the “minimal geometric mantle” but there is no proof that this is “minimal” in a strict mathematical sense. Moreover, as noted in point 3, a “mantle” is never defined in this context. Further, there are no references to existing geometric techniques or established methods (either to compare or contrast to).
7. It’s not clear why this new geometric method is even necessary, given that most of the information it detects is reproduced by PH (as stated in the Results section). In the presence of two methods that provide mostly the same information, we should prefer the well-established, mathematically rigorous one. If it is the case that the geometric method provides additional significant biological information, that should be explicitly noted.
8. The most striking issue, in my opinion, is the repeated claim that the authors have developed both the geometric and topological methods. In the case of the topological method PH, this is simply not true. It is not clear to me what the authors are specifically referring to regarding their novel contribution to this long-established topological method. This must be clarified.
9. The most compelling piece of this paper is their description of why a topological or geometric investigation of the islets of Langerhans would be important. Yet, in the discussion, the contribution of the authors is simply noting the differences in cell composition across developmental stages. The authors even note that this fact has been established by previous studies. Given this, it is not clear what the biological contribution of this work is.
