# Supplementary material for: Geometric and topological characterization of the cytoarchitecture of islets of Langerhans
Source: PLoS Comput Biol. 2023 Nov 9;19(11):e1011617. doi: 10.1371/journal.pcbi.1011617 (PMC10662755; doi:10.1371/journal.pcbi.1011617)
Supplement: Attachment — Submitted filename: Islet_paper_rev1.docx [file pcbi.1011617.s018.docx]

**Reviewer 1**

Geometric and Topological Characterization of the Cytoarchitecture of Islets of Langerhans

The article deals with a geometric technique and persistent homology (PH) for the investigation of the cytoarchitecture of the islets of Langerhans.

Minor concerns:

1. PH is described as computing topological invariants “from networks,” which is incorrect. PH computes topological invariants from a simplicial complex upon a point cloud of data.
2. There are repeated references to PH uncovering “non-local robust network features,” but this is never defined. It is unclear what “non-local” refers to and what sort of robustness is used.
3. Their explanation of PH in Methods discusses vertices, edges, and triangles, but makes no mention of higher-dimensional structures that may appear in the simplicial complex. These structures can appear even in lower-dimensional data due to the construction of the simplicial complex. Additionally, the words used (borrowed from network theory and geometry) are not the proper vocabulary for these topological objects. In the context of PH, these are the 0-simplex, 1-simplex, 2-simplex, etc.
4. The description of the PH methods are very technical and difficult to parse. I would suggest referring to existing explanations of PH in the literature to see if it can be simplified for a computational biology audience.

We deliberately toned down the mathematical terminology to make it accessible to audience of PLOS computational biology where there are very few experts in algebraic topology. This seems to have led to the minor concerns of the reviewer who clearly is an expert. We are not sure that we are capable of simplifying the explanation of PH methods while using the precise terminology. Several experts in the field have written explanations and we now include annotated references (Fugacci et al Persistent homology: a step-by-step introduction for newcomer, 2013 and Bauer Ripser: efficient computation of Vietoris--Rips persistence barcodes, 2021).

Major concerns:

1. PH is computed using software developed by one of the authors called Dory. While information regarding Dory has been uploaded to arXiv, the software has not been published in a peer-reviewed setting. This raises questions about the accuracy of Dory compared to published software like Ripser, giotto-tda, javaPlex, etc.

We have removed Dory from the manuscript. PH is computed by a straightforward implementation of the standard column algorithm (in C) mentioned in Methods. Same results can be reproduced using Ripser (Bauer, JACT 2021) which is computationally more efficient in runtime and memory usage as compared to a straightforward implementation of the standard column algorithm.

2. There is a claim that the authors have developed “technical tools for finding tight representatives for topological features that can be explicitly localized for visualization.” It is not clear to me what this means. The discussion further claims that the authors’ method “explicitly give[s] the locations of the actual topological features in the image,” but no such method is described in the Methods section or described in the Results.

The strategy to find tight representatives was introduced in Aggarwal M, Periwal V (2023) Tight basis cycle representatives for persistent homology of large biological data sets. PLOS Computational Biology 19(5): e1010341. We have clarified this in the manuscript now and added the citation.

“Mantles” are computed using geometric and topological techniques but are not defined.

We define ‘mantle’ in Introduction now as cycles of cells (close to the periphery) around cores of cells of the opposite type. Since we find cycles that can be anywhere in the islet, we have replaced ‘mantle(s)’ by ‘cycle(s)’ in the text.

3. Figure 5B features two separate line graphs consisting of two datapoints each (control vs. diabetic), which is not an appropriate visualization for this data. There is no statistical test to compare the percentiles between control and diabetic.

Supplementary Figure S2 shows comparison of box-plots of distribution of maximum persistence and statistical significance using Mann-Whitney U test shows that they are significantly different. The percentiles are shown in the main text to highlight correlation in trends between persistence and cycles computed.

We have now added box-plots of distribution of maximum persistence for both developmental data set as Supplementary Figure S9.

4. A pair distribution function is used for the distance computation in PH. It’s not immediately obvious to me that the function is a metric. The construction of a simplicial complex requires a metric. Please provide a reference or proof to establish this.

The pair distribution function is only used to estimate the threshold of pairwise distances to define the final graph (or simplicial complex in PH). The pairwise distances are Euclidean metric.

5. The geometric method introduced is complex and the significance of each step is not explained. It is claimed that this method detects the “minimal geometric mantle” but there is no proof that this is “minimal” in a strict mathematical sense. Moreover, as noted in point 3, a “mantle” is never defined in this context. Further, there are no references to existing geometric techniques or established methods (either to compare or contrast to).

We have re-written the geometric method in Methods. Specifically, we explicitly mention that our geometric method picks the minimal counterclockwise turn at every node along the cycle. This minimizes the local curvature by construction, and therefore results in a geometrically smooth cycle.

The geometric cycles obtained using this minimality criterion are geometrically close to the shortened representative homology cycles, as shown in Results. This is not mathematically obvious, to the best of our understanding.

The core-mantle structures have previously been studied by visual inspection of islet slices (Bosco et al, Unique arrangement of alpha and beta cells in islets of Langerhans, Diabetes 2010). Rigorous mathematical definitions of similar ring structures for islet cytoarchitecture and algorithmic implementations thereof were lacking.

6. It’s not clear why this new geometric method is even necessary, given that most of the information it detects is reproduced by PH (as stated in the Results section). In the presence of two methods that provide mostly the same information, we should prefer the well-established, mathematically rigorous one. If it is the case that the geometric method provides additional significant biological information, that should be explicitly noted.

We are doing this in two different methods because this has been a subject of controversy for decades and biologists prefer visual representations which the geometric method provides. On the other hand, the PH method can generalize to 3D data which is difficult for the geometric method. We now illustrate cycles found in 3D data sets of islets in Results.
The geometric method can inform us about cores of cells that are surrounded partially by cells of the other type. We note this explicitly in the text and provide an example as a Supplementary figure. Analysis of partial loops around cores may be useful across developmental stages, but it is not in the scope of this manuscript.

7. The most striking issue, in my opinion, is the repeated claim that the authors have developed both the geometric and topological methods. In the case of the topological method PH, this is simply not true. It is not clear to me what the authors are specifically referring to regarding their novel contribution to this long-established topological method. This must be clarified.

We clarify it in the last paragraph of Introduction now.

Briefly, representative boundaries around topological features are not unique (by definition) and can be geometrically imprecise. To ascertain possible functional significance of these cycles by analyzing their properties, it is important that precise representative boundaries are computed. In Aggarwal M, Periwal V (2023), we developed tools to compute representative boundaries with higher geometric precision. In this work we define any alpha-delta cycle that contains beta-cells inside (and vice versa) as biologically significant and introduce a method to quantitate all biologically significant topological features. This contrasts with the traditional topological data analysis using PH that classifies topological features as significant based on a threshold for mathematical persistence.

8. The most compelling piece of this paper is their description of why a topological or geometric investigation of the islets of Langerhans would be important. Yet, in the discussion, the contribution of the authors is simply noting the differences in cell composition across developmental stages. The authors even note that this fact has been established by previous studies. Given this, it is not clear what the biological contribution of this work is.

This work enables large-scale analysis of 2D and 3D islet structural information that can be used to address controversies and debate importance of ring structures that have been observed by visual inspection previously.

The contribution to biology is only to provide quantitation of structures that have been controversial in terms of existence and/or functional significance. We hope that by providing multiple mathematical methods for defining and computing such topological structures, the field can focus on the relevance of these structures for function. We have stated this explicitly in the discussion.

**Reviewer 2**

This article takes a dual geometrical and then topological view of islet architecture to consider the impact of non-local cellular interactions (e.g. cycles of delta cells around beta cells, spherical enclosure of beta cells around alpha cells).

The authors extend their previous work on islet graph analysis to account for mantle identification. They describe in good detail the algorithm used and inspire confidence in their method.

They show a significant disruption in the mantle structure of type 2 diabetes islets, which agrees with previous results using other measures.

I think this is a valuable and technically sophisticated extension of topological methods to islet structure/function discussions.

There are a few questions, suggestions, and minor edits:

Code to generate the results (or similar) should be published for reproducibility.

The code is available at https://github.com/nihcompmed/Pancreatic-Islets.

Topologies that are considered appear to be fundamentally two-dimensional which may not always be an appropriate reduction (e.g. untrapped from a cycle in a third dimension). While the ability to discuss 3D structure is clear in the method, the authors do not seem to venture an example where 3D data is involved? Can such an example, say from slice (or other) data, be included to support the applicability in 3D?

We implemented computation of cycles in 3D islets and have explained it in Methods. We analyzed data sets for human (n=28) and mice (n=29) that were publicly available from Hoang et al 2014. We show a few examples in Results.

Can you confirm enclosure of NS components (i.e. while centers of cells may appear to enclose components perhaps the boundary could be breached by an oddly shaped neighboring cell)? Are there examples of data with cell membrane markers included to tesselate the space and confirm mantle enclosure that is suggested by cell centers? This also relates to how "Edges obstructed by a cell are removed"(pg 7) Is there a certain tolerance for edge removal(i.e. if the obstructing cell center is within 1-5 microns along say the orthogonal projection to the edge, then the edge is removed)?

This is absolutely true, delta cells have electrical signals between cells. We do not have the data for this scenario.

The edges obstructed by a cell are removed using ‘shadow algorithm’. Briefly, neighborhood radius is first determined using the correlation function. Then, for a given cell, its neighbors are computed and sorted by their distance from the cell. This list of neighbors is traversed and edge from the cell to a neighbor is not considered if the neighbor is in the ‘shadow angle’ of any of the previous cells in the list of neighbors. The computation of the shadow angle assumes radius of the cell to be 4 $\mu$m. This is done for each cell. An illustrated example can be found in Figures 1D and 1E in Striegel el at, The Beta Cell in Its Cluster, 2015.

Is it surprising that so low (649/6088, 463/4942, 168/3130, and 453/7203) of fractions of islets at the four stages (assuming it is the same data sets/numbers shown in Fig 3) had an alphadelta-mantle with NS beta-component? Around 10% (or less for later stages)? Does this undermine the importance of these structures in islet function? Or are there other features that might act to prevent connection of mantles in the calculation but yet yielding de facto mantle-like function (e.g. Blood vessels, etc) and increasing the effective mantle structures in reality?

Even if low in percentage, we observe trends in the topology across development that correlate with the percentage of islets with mantles (Figure 3). It is possible that these changes in the small percentage of islets are functionally relevant.

We now mention in the discussion that our results are correlative not causative and how these structures are related to glucose stimulated insulin secretion (GSIS) profiles is unclear. Our aim is to quantitate these structures so that when we understand how different features in the GSIS profile are related to specific islet features, then this quantitation might be useful.

The reviewer is exactly on point, blood vessels can play a role in mantle-like function. Data sets with blood vessel information are just becoming available (Manami Hara et al, Pancreatic regional blood flow links endocrine and exocrine diseases, JCI 2023). We are in the process of thinking of how to incorporate this information in cycle computation.

An aggregate analysis made up of the known stages (Figs 2-4) might be useful to then connect with the control and T2D data where you are without stage information. For example, does the control data look like the combination of the data from Figs 2-4? If not, would you be able to predict/suggest what stage (or combination of stages) the control data most resembles?

The T2D data set is from adult human islets (minimum age 15 years and median age 64 years) from deceased subjects. It might not relate to the aggregate of the developmental stages. We have added demographics of the subjects in the T2D data set in Supplementary Figure S8.

The number of samples in developmental and control are very different in the current data sets to comment on statistical significance of any correlation that may be present by chance.

Is it useful to apply your analysis to an example with mice or rat data data with more straightforward core and mantle geometry?

It would potentially be useful to apply to mice. We do not have access to detailed mouse data for cell composition. This data is very rare at this quantity of islets.

The authors mention stochastic shortening in mantle calculation of PH-mantles, but not clear where this was utilized in the calculations.

We have restructured Methods and clarified where and why shortening of PH-mantles is needed.

Minor edits:

The authors mention insulin and glucagon in first part of abstract, but then mention delta cells in geometry. Is somatostatin an important regulator or is the electrical connections with delta cells more important? Alpha and delta cells are merged in the analysis, but a more parallel introduction would read better.

Electrical connectivity of delta cells is just being appreciated in recent research (see Dou et al, Optogenetic Control of Pancreatic Delta Cells Reveals Their Central Role in Hormone Release from Pancreatic Islets, Diabetes 2023).

When implementing the dirac delta function as a practical matter, is there a tolerance (i.e. cells with a distance of r +/- 1% of r are counted as being at a distance r away)? (pg 7)

The pair distribution function is computed as ratio of number of cells at a radial distance of r to the number of cells expected if they are randomly distributed. We have revised the manuscript with this definition. The thickness of the radial shells was chosen as dr = 0.5.

Geometry subscript on beta-graph is denoted by alphadelta rather than beta. (pg 7)

Corrected, thank you.

Adding a "Stage x" (x being 0, 1, 2, or 3) in white in the upper right corner of each of the four graphs in Fig 2 (and similar latter graphs) would help. Even though the authors were explicit in the caption where to start stage 0 I still took it as the positive x-axis (East rather than West) to start.
Labeling the columns (control vs. diabetic) in Fig 5 would support ease of comparison.

Figures have been modified according to both suggestions.

Reviewer #3: Please find below my minor and major comments organized in a systematic manner. I think your article is potentially of great interest to the beta cell community and other fields dealing with similar problems of quantifying architectural features in a robust manner on large data sets. However, I think the article could benefit a lot by taking into account my suggestions and comments.

Minor
Introduction

Thank you for the very detailed constructive suggestions and comments.

1-2 % of islet mass may be a slight underestimation, please see Dolensek et al Islets 2015 for more data.

Replaced 1-2% by 1-4% of islet mass as mentioned in Dolensek et al and added citation.

Islets secrete hormones that are crucial for regulating blood glucose levels, as well as levels of amino acids, free fatty acids, keto acids, glycerol, and other energy-rich nutrients. Given the often too glucocentric view on islets and diabetes, perhaps glucose could be replaced by energy-rich nutrients.

Added ‘as well as levels of amino acids, free fatty acids, keto acids, glycerol, and other energy-rich nutrients.’ (verbatim)

In second paragraph of the introduction, the influence of insulin on glucose is pointed out, but its effects extend beyond that, to storage of glucose in the liver in the form of glycogen, as well as to promoting storage of lipids in fat tissue (inhibition of the hormone-sensitive lipase, promotion of lipoprotein lipase), and amino acids in muscle tissue.

Added ‘storage of glucose in the liver in the form of glycogen, as well as to promoting storage of lipids in fat tissue (inhibition of the hormone-sensitive lipase, promotion of lipoprotein lipase), and amino acids in muscle tissue’ (verbatim) as effect of insulin to introduction.

In the second or third paragraph of the introduction, it may be worth mentioning that the relationship between islets and acinar and ductal cells may also be different at different developmental stages and in different species (e.g., islets are more intralobular in humans and more interlobular in mice).

Added ‘The relationship between islets and acinar and ductal cells may also be different at different developmental stages and in different species (e.g., islets are more intralobular in humans and more interlobular in mice).’ (verbatim)

In paragraph 4, I think it is important to mention that early during development of diabetes there may be an adaptive increase in mass or function (or both) and that in some people (non-progressors), this may be more functional or persistent than in others (progressors) and is crucial for our understanding why some people do not develop T2D despite insulin resistance and why some respond to dietary interventions while others don´t. See for instance Taylor et al Cell Metab 2018, Stozer et al Nephron 2019, Boland et al Mol Metab 2017, Cohrs et al Cell Rep 2020, Hudish et al JCI 2019, Weir&Weir Diabetes 2004.

Added ‘Early during development of diabetes there may be an adaptive increase in mass or function (or both) and that in some people (non-progressors), this may be more functional or persistent than in others (progressors) and is crucial for our understanding why some people do not develop T2D despite insulin resistance and why some respond to dietary interventions while others don´t.’ (verbatim)

2 Results
Has the developmental data set or part thereof been used before in previous publications? If yes, please specify.

We have specified it now in Results.

What were the donor characteristics for control and T2D human islets and for developmental donors? If available, a table summarizing the main characteristics would be much appreciated. If not, please point out that the data are not available.

We show donor characteristics for T2D data set in Figure S8 and refer to it in Results first paragraph. Demographics for developmental data set was not available.

Why do the authors combine alpha and delta cells into the alpha-delta group? How does this affect the results?

Alpha and delta cells were combined because some previous studies reported that they are found in mantles together (Orci and Unger, Functional subdivision of islets of Langerhans and possible role of D cells. Lancet 1975).

We analyzed the ratio of alpha to delta cells in both data sets and found that later stages of development have higher proportion of islets that have more alpha than delta cells (Figure 2B). Both control and diabetic subjects also have a large proportion of islets with more alpha than delta cells (Supplementary Figure S10), reasonably because most of these islets are from adult human subjects.

We conducted a comparison between alpha mantles and alpha-delta mantles as follows. Alpha mantles around beta cells were computed. We show the counts of islets with at least one alpha-mantle that contains at least one beta-cell below. In parenthesis we show the number of islets with at least one alpha-delta mantle that contain at least one NS beta-component that are reported in the manuscript.

Stage 0: 139 (629, ratio 139/629= 0.22)
Stage1: 103 (439, ratio 103/439= 0.23)
Stage2: 61 (165, ratio 61/165= 0.37)
Stage3: 346 (440, ratio 346/440= 0.78)
Control: 115 (175, ratio 115/175= 0.65)
Diabetic: 71 (85, ratio 71/85=0.83)

It can be reasoned that the ratio of alpha mantles to alpha-delta mantles is higher for stages 2,3, Control, and diabetic because these categories have large proportion of islets with more alpha than delta cells, making the effect of ignoring delta cells in mantle-computation less significant.

Please explain the abbreviation NS components.

We have added explanation of the abbreviation in the first paragraph of Results.

Figures 2, 4, and 5 would be easier to read with stages (T2D status) indicated also on figures (there is enough space in the top black section to add stage 0, stage 1 etc. Additionally, for the more old-fashioned beta cell physiologists, some exemplary/representative 2D sections for each stage (and CTRL vs T2D) next to KDE plots would be much appreciated.

We have modified the figures accordingly. We show exemplary 2D sections as supplementary figures (S11 and S12).

Discussion
Functional connectivity seems to be reduced under diabetogenic conditions (islets cultured in high glucose with or without palmitate, islets incubated in cytokines, islets from T2D donors) and this has been explicitly or implicitly attributed to decreased gap-junctional connectivity or disrupted cytoarchitecture (e.g., vessels disrupting beta cell syncytium). See for instance work by Richard Benninger, Guy Rutter, David Hodson, and Andraz Stozer. Do your findings of less mantles in T2D islets comply with these findings? Does the lower number of mantles in any way support the view that the beta cell syncytium may be less well connected, that there may be more bottlenecks for intercellular waves spreading between cells? Please include a short paragraph on the relationship between your morphological and these functional findings.

We have added a discussion on this in third paragraph of Discussion.

Major
Are for any of the data sets 3D stacks of 2 D sections available? If yes, it would be a great added value to show to what extent the computed/determined characteristics are stable or heterogeneous within islets, i.e., from one pole, over the equatorial plane, and to the other pole. I reckon that such analysis would also enable the authors to determine whether the mantles can be found at all parts of an islet or preferentially closer to the poles or the equatorial plane. I understand that such sections may not be available. In that case, the authors could briefly point this out as a drawback and suggest some ideas for further studies and analyses.

We implemented computation of cycles in 3D islets and have explained it in Methods. Data sets for human (n=28) and mice (n=29) that were publicly available from Hoang et al 2014 were analyzed. A few examples are shown in Results.

In the limited 3D data sets that were available, we observed islets with highly convex shapes and some with multiple globules. Mathematically defining and computing equatorial plane and poles for these 3D point-clouds does not seem to be trivial. It will require a larger number of data sets to test and validate an algorithm to compute these geometrical properties. Once we can define and implement this computation and test it for many 3D islets, it will be of interest to compare heterogeneity between different slicing schemes. We mention this in the discussion now.

Additionally, for the existing 2D data set, I think it would be of great value to include an analysis of the positions of the mantles found in islets with respect to islet center/periphery.

We have added this analysis to Results and a corresponding section in Methods.
